# Editorial: Advances in Optical Elastography

**DOI:** 10.1117/1.JBO.30.12.124501

**Published:** 2025-12-13

**Authors:** Stefan Catheline, Irina Kabakova, Kirill V. Larin

**Affiliations:** a University of Lyon, Department of Health, Lyon, France; b University of Technology Sydney, School of Mathematical and Physical Sciences, Sydney, Australia; c University of Houston, Department of Biomedical Engineering, Houston, Texas, United States

## Abstract

The editorial introduces the JBO Special Section “Advances in Optical Elastography” for Volume 30 Issue 12.

Optical elastography continues to advance rapidly, offering noninvasive, high-resolution insight into the mechanical properties of biological tissues. By combining the structural imaging capabilities of optical coherence tomography and related modalities with quantitative elasticity mapping, optical elastography has become a vital tool for exploring the biomechanical basis of disease. The papers collected in this JBO Special Section on Advances in Optical Elastography reflect the field’s growing technical sophistication and translational potential, spanning methodological innovation, validation through realistic phantoms, and diverse clinical applications.

## 1 Emerging Methods for High-Speed and Quantitative Elastography

Several contributions in this issue highlight innovations designed to increase imaging speed, motion tolerance, and measurement precision. Soundararajan et al.^1^ introduced a retrospective, respiratory-gated anatomical optical coherence tomography approach for airway wall elastography, enabling cross-sectional compliance measurements over a 50-mm airway segment in under 1 min. Their 4D sawtooth scanning strategy allows precise mapping of airway deformation during tidal breathing and represents an important step toward clinical airway elastography during bronchoscopy.

Schmidt et al.^2^ reported an asynchronous optical coherence elastography (OCE) technique that operates at conventional OCT acquisition rates while providing robust three-dimensional shear wave imaging (featured on the issue cover). Combined with their new directional phase gradient analysis method, this approach enables accurate wave number estimation under a variety of propagation conditions—without hardware modification.

Regnault et al.^3^ extended the theoretical foundations of dynamic OCE to nearly incompressible, transversely isotropic (NITI) media. They demonstrated accurate reconstruction of tensile and shear moduli using Rayleigh wave phase velocities and cautioned that commonly used group velocity analyses can lead to erroneous moduli inversion. Their framework establishes a rigorous pathway for quantitative elastography of anisotropic tissues such as muscle, skin, and cornea.

Complementing these developments, Xu et al.^4^ proposed a reverberant OCE approach employing 3D-printed, randomly distributed scatterers to enhance displacement field uniformity in hydrogel cultures. Their work showed that optimized scatterer distributions yield highly diffuse reverberant fields, improving elasticity estimates that align closely with shear rheometry.

## 2 Realistic Phantoms and Model Systems

Navaeipour et al.^5^ advanced phantom development by fabricating breast-mimicking phantoms that reproduce both the microstructural and mechanical features of normal and cancerous breast tissue. Using 3D-printed molds and a dissolving-mold technique, they successfully replicated ductal and carcinoma-like architectures down to 100  μm. These models provide a valuable platform for evaluating OCE performance and investigating how complex tissue morphology influences elastogram formation.

## 3 Expanding Biological and Clinical Applications

The growing clinical relevance of OCE is evident in several contributions that explore its application to living and *ex vivo* tissues. Singh et al.^6^ applied noncontact, air-coupled ultrasound OCE to quantify the stiffness of murine bone marrow and reported significant age-related increases in elastic modulus. The same group demonstrates that wave-based OCE can detect elevated corneal stiffness in a nonhuman primate model of glaucoma, correlating mechanical remodeling with chronic intraocular pressure.^7^

Yang et al.^8^ presented a piezoelectric-transducer-based OCE system for *in vivo* assessment of oral submucous fibrosis. Their results show a more than fourfold increase in tissue stiffness in fibrotic compared with normal mucosa, illustrating OCE’s diagnostic potential for early, noninvasive fibrosis detection.

Schumacher et al.^9^ introduced a multimodal Brillouin–OCE system for *in vivo* lens biomechanics. The platform combines Brillouin spectroscopy with wave-based OCE on a slit-lamp interface, enabling sequential imaging of porcine and human lenses. Human measurements showed age-related stiffening, with OCE wave speeds nearly doubling compared to porcine eyes, while Brillouin profiles revealed depth-dependent stiffness variation.

## 4 Mechanobiology and Brillouin Microscopy

The section concludes with a comprehensive review by Falkner et al.^10^ on the role of Brillouin microscopy in cancer mechanobiology. This label-free optical elastography technique probes viscoelasticity through light scattering from acoustic phonons. The authors synthesized findings across cell cultures, organoids, and tissues, showing that Brillouin imaging can consistently distinguish malignant from healthy samples and track mechanical changes during cancer progression and treatment. The review also identifies key challenges for clinical translation, including measurement standardization, artificial-intelligence-assisted data analysis, and *in vivo* hardware adaptation.

## 5 Outlook

Collectively, the contributions in this special section underscore the maturation of optical elastography from experimental imaging to translational science. Improvements in acquisition speed, motion robustness, and quantitative modeling now converge with realistic tissue phantoms and clinically relevant validation. Integration with complementary modalities such as Brillouin microscopy promises a comprehensive biomechanical view of tissues across scales—from cellular mechanobiology to organ-level pathology.

As these papers demonstrate, optical elastography continues to expand the frontiers of biomedical optics, enabling new opportunities in diagnostic imaging, disease monitoring, and therapeutic evaluation. We extend our gratitude to the contributing authors and reviewers for their efforts, and to the *Journal of Biomedical Optics* for supporting this special issue highlighting the next generation of optical elastography research.
